# Learning lessons from field surveys in humanitarian contexts: a case study of field surveys conducted in North Kivu, DRC 2006-2008

**DOI:** 10.1186/1752-1505-3-8

**Published:** 2009-09-10

**Authors:** Rebecca F Grais, Francisco J Luquero, Emmanuel Grellety, Heloise Pham, Benjamin Coghlan, Pierre Salignon

**Affiliations:** 1 Epicentre, 8 rue Saint Sabin, 75011 Paris, France; 2 European Programme for Intervention Epidemiology Training, European Centre for Disease Prevention and Control, Stockholm, Sweden; 3 Centre for International Health, Burnet Institute, Melbourne, Australia; 4 Health and Nutrition Tracking Service, Geneva, Switzerland

## Abstract

Survey estimates of mortality and malnutrition are commonly used to guide humanitarian decision-making. Currently, different methods of conducting field surveys are the subject of debate among epidemiologists. Beyond the technical arguments, decision makers may find it difficult to conceptualize what the estimates actually mean. For instance, what makes this particular situation an emergency? And how should the operational response be adapted accordingly. This brings into question not only the quality of the survey methodology, but also the difficulties epidemiologists face in interpreting results and selecting the most important information to guide operations. As a case study, we reviewed mortality and nutritional surveys conducted in North Kivu, Democratic Republic of Congo (DRC) published from January 2006 to January 2009. We performed a PubMed/Medline search for published articles and scanned publicly available humanitarian databases and clearinghouses for grey literature. To evaluate the surveys, we developed minimum reporting criteria based on available guidelines and selected peer-review articles. We identified 38 reports through our search strategy; three surveys met our inclusion criteria. The surveys varied in methodological quality. Reporting against minimum criteria was generally good, but presentation of ethical procedures, raw data and survey limitations were missed in all surveys. All surveys also failed to consider contextual factors important for data interpretation. From this review, we conclude that mechanisms to ensure sound survey design and conduct must be implemented by operational organisations to improve data quality and reporting. Training in data interpretation would also be useful. Novel survey methods should be trialled and prospective data gathering (surveillance) employed wherever feasible.

## Introduction

In media and agency reports on complex emergencies, an estimate of the number of people who have died, the prevalence of childhood malnutrition and other key health indicators are often quoted. Although a discriminating reader may understand that these are estimates, we rarely question how or from where these numbers come. In most cases, estimates are obtained by means of field surveys which are subject to a number of limitations. In the past, the application of standard survey methods by various humanitarian actors has been criticised [1]. Currently, different methods of conducting field surveys are the subject of debate among epidemiologists and their strengths and weakness have been described in the literature [2-6]. Beyond the technical arguments, decision makers may find it difficult to conceptualize what the estimates actually mean. For instance, what makes this particular situation an emergency? And how should the operational response - humanitarian, political, even military - be adapted accordingly [7,8]? This brings into question not only the quality of the survey methodology, but also the difficulties epidemiologists face in interpreting results and selecting the most important information to guide operations.

**Figure 1:**
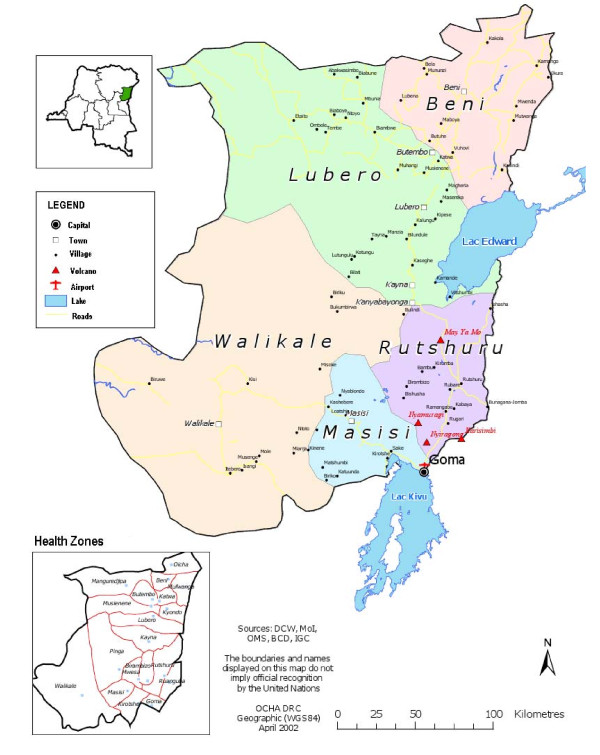
**Map of North Kivu, Democratic Republic of Congo**.

As a case study, we reviewed publicly available field surveys of a current acute-on-chronic humanitarian crisis - North Kivu, Democratic Republic of Congo (DRC) - to examine the methodologies employed, the findings presented, the interpretation of the results and the recommendations made. The eastern DRC Province of North Kivu has been the scene of conflict that has erupted sporadically for over a decade (Figure 1). The most recent renewal of violence has forced some 250,000 people to flee their homes since August 2008 [9].

## Methods

We searched PubMed/Medline for articles published from January 1, 2006 to January 1, 2009, in English, French, German, and Spanish using the key words ["mortality" (major topic) OR "nutrition" (major topic)] AND ["Congo" (text word) OR "Democratic Republic of Congo" OR "North Kivu"]. To identify non-peer-reviewed reports, we performed the same search in: (i) the Human Impact of Complex Emergencies Complex (CE-DAT) database; (ii) Relief-Web (a media and NGO repository maintained by the Office for the Coordination of Humanitarian Affairs); (iii) RDC-humanitaire.net; and (iv) the websites of selected large international NGOs (the International Rescue Committee, Merlin, Action Contre la Faim, UNICEF, UNHCR, and Médecins Sans Frontières). We also contacted the individual organizations above and also requested additional information from the Health and Nutrition Tracking Service (HNTS).

Inclusion criteria were a written report with, at minimum: 1. an estimate of the crude mortality rate (CMR); 2. the under five mortality rate (U5MR); and 3. the prevalence of global (GAM) and severe acute malnutrition (SAM) in the surveyed population. We excluded meta-analyses, commentaries, reports on DRC with no specific information about North Kivu, multi-sector agency evaluations not based on a survey, humanitarian action plans and rapid assessments of small or non-randomized populations. We drew from criteria proposed by Mills et al. [10], Checchi and Roberts [11], the STROBE guidelines [12] and the SMART initiative [13] to review the publications and to propose a standard reporting format for field surveys. Review criteria included those common to the published work [10-13] in addition to drawing from the authors experiences.

## Results

**Figure 2:**
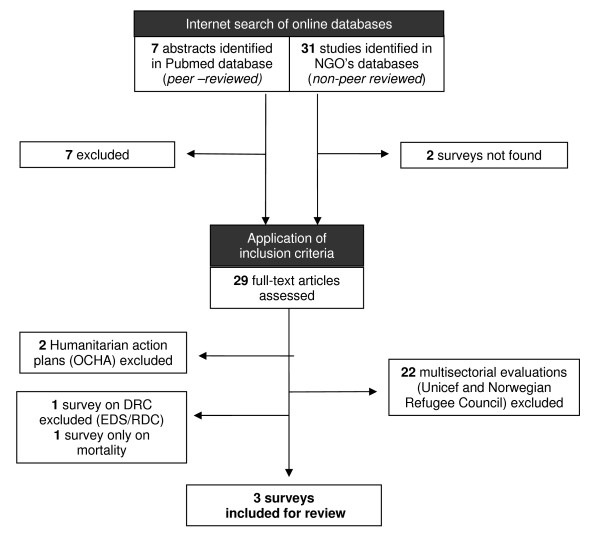
**Flow diagram of surveys included in the analysis**.

We identified 38 agency reports through our search strategy (Figure 2): seven from PubMed/MEDLINE, four through CE-DAT, one through Reliefweb, 23 from RDC-humanitaire.net, and three via individual web-sites. No additional reports were identified through citations. We were able to obtain 36 of the 38 reports. (The two documents we could not source were a rapid field assessment conducted by Action Contre la Faim in November 2008, and a nutritional survey conducted by World Vision in Rwanguba health zone in March 2007.) Only three of the 36 surveys met our inclusion criteria. We excluded 22 multi-sector evaluations, two humanitarian action plans, one survey covering the entire country but without specific mention of North Kivu, and one country-wide survey of mortality without a nutritional assessment.

All three surveys were conducted by respected international non-governmental organizations (NGOs) during 2008: Action Contre la Faim (ACF) [14], Cooperazione Internazionale (COOPI) [15] and Epicentre [16]. All reported similar results for CMR, U5MR and prevalence of malnutrition (see table 1 and table 2), and all assessed measles vaccination coverage. Two of the studies also assessed other health indicators.

**Table 1:** Description of methodology for reviewed surveys

**No.** **(Ref)**	**Time**	**Place**	**Rationale**	**Objectives**	**Method**	**Population**	**Recall period for mortality**
**1 (14)**	June 2008	Kibua	◦ This is the first mortality and nutritional assessment performed in Kibua (performed by this NGO).	◦ To estimate the prevalence of acute and chronic malnutrition among children 6-59 months ◦ To determine the crude and the under five mortality rate ◦ To estimate the measles vaccine coverage ◦ To estimate the vitamin A supplementation coverage ◦ To assess the deparasitation among children with Mebendazol	Two-stage household based cluster sampling	81,174	90 days

**2** **(15)**	July 2008	Binza	◦ The NGO implemented a nutritional program in 2008 and provides technical, financial and material support to the nutritional centers operated by a national NGO.	◦ To estimate the prevalence of acute and chronic malnutrition among children 6-59 months ◦ To determine the crude and the under five mortality rate ◦ To estimate the measles vaccine coverage ◦ To estimate the vitamin A supplementation coverage ◦ To assess the feeding practices among young children	Cluster based sampling	102,284	90 days

**3** **(16)**	July-August 2008	Nyanzale, Birambizo	◦ To provide humanitarian aide adapted to the displaced population ◦ Follow the heath situation in the displaced camps in the region of Nyanzale	◦ To assess the mortality rate ◦ To assess the nutritional status of the children ◦ To evaluate the measles vaccination coverage ◦ To implement a mortality surveillance system	Systematic sampling	1701 households	60 days

**Table 2:** Description of results and recommendations for reviewed surveys

**No.** **(Ref)**	**Time**	**Place**	**CMR** **(per 10,000 per day)**	**U5MR** **(per 10,000 per day)**	**GAM** **(WHO)**	**SAM** **(WHO)**	**Recommendations**
**1** **(14)**	June 2008	Kibua	0.38 [0.18-0.58]	1.10 [0.45-1.76]	4.8% [3.2-6.3]	0.5% [0.1-1.0]	◦ Community awareness about key themes in nutrition and encourage them to visit the NGO for preventive consultations ◦ Support the implementation of food security assessment to improve food production and diversity ◦ Put in place a nutritional education system ◦ Reinforce routine vaccination activities ◦ Put in place comprehensive management of acute malnutrition in health centers. ◦ Improve the sources of potable water

**2** **(15)**	July 2008	Binza	0.53 [0.30-0.76]	0.88 [0.25-1.51]	5.1% [3.3-7.0]	1.0% [0.3-1.7]	◦ Continue activities for moderate malnutrition to prevent the risk of severe malnutrition ◦ Provide breastfeeding and nutritional counselling ◦ Support routine vaccination activities and distribution of vitamin A ◦ Improve the overall health status of the population

**3** **(16)**	July-August 2008	Nyanzale, Birambizo	0.48 [0.22-1.05]	1.08 [0.37-3.14]	2.6% [1.4-4.5]	0.9% [0.4-2.4]	◦ Repeat the nutritional assessment the following year at the same time using the same methodology ◦ Active screening of children's nutritional status ◦ Put in place a prospective surveillance system for morbidity and mortality ◦ Strengthen routine measles immunization strategies ◦ Alert authorities to an abnormal increase in the number of cases of malaria, diarrhea and measles ◦ Community awareness campaign about the NGOs activities

## Discussion

While three surveys is a small sample to review, several important lessons can still be learned about how field surveys should be conducted, how they should be reported, and what they should be expected to achieve. First, although surveys may be designed by seasoned field epidemiologists, many are performed by less qualified and experienced staff which can lead to methodological shortcomings. For example, one survey sampled the first 30 households at the center of each cluster, a mistaken application of the WHO EPI survey methodology [17] biasing their sampling. Such errors waste limited resources and can result in programmatic decisions based on misleading data. Currently, there is no formal mechanism for organizations to have survey protocols reviewed - which may mean protocols do not even get written. Ethical approval may be routine practice for many organizations to prevent harm to participants, but there remains no adequate means to discuss survey design, survey instruments or even concerns about the need for surveys. Such technical and contextual issues may not be well understood by ethical review boards, but may certainly impact on the ethics of conducting the study. Having experienced staff review survey protocols before data collection begins can improve the chances that surveys will provide informative data. More formal review of surveys meant for advocacy purposes can help ensure they will be met with greater acceptance. The recently formed Expert Review Group of the HNTS, or another similar body, such as the Technical Advisory Group of SMART, could be suitable bodies for peer-review of protocols if accomplished in a timely manner. This would go some way to helping prevent the conduct of substandard (and consequently unethical) surveys and improve the overall quality of information collected.

Unlike other areas of epidemiology, for example, the CONSORT [18] and STROBE [12] guidelines for clinical trials and observational studies, there are no standardized reporting guidelines for field surveys in humanitarian contexts. Reporting standards offer a way for epidemiologists to prepare survey reports, improve transparency, and facilitate critical appraisal and interpretation. The Standardized Monitoring and Assessment of Relief and Transitions (SMART) initiative aims to ensure standardization of planning, training, analysis and reporting [13], and advocates for the systematic use of mortality and nutrition indicators. The evaluation criteria presented in table 3 and table 4, is a first step towards developing a checklist for field surveys conducted in humanitarian contexts. For the three surveys we reviewed, reporting of ethical considerations, procedures for dealing with empty households, raw data and survey limitations were commonly missed. Follow-up actions for using the information were lacking for two of the three studies. In general, however, the three surveys we reviewed fulfilled most of the criteria.

**Table 3:** Critical review criteria (background and methodology) and results of three reviewed surveys

	**Survey** **(Ref)**			
	**1** **(14)**	**2** **(15)**	**3** **(16)**	
				**Criteria**

**Background**				

Rationale	✓	✓	✓	Explain the rationale for the survey
Objectives	✓	✓	✓	State the objectives
Utilization	✓	✓	✓	State how the results of the survey are to be used (e.g. advocacy, program monitoring, baseline assessment)
Protocol	✓	✓	✓	State who wrote the protocol for this survey

**Methods**				
Setting	✓	✓	✓	Describe the survey setting and relevant dates
Participants	✓	✓	✓	Give the eligibility criteria for inclusion in the survey
	✓	✗	✓	State the definition of household
Variables	✓	✓	✓	Define all outcomes and exposures
Survey instrument(s)	✓	✓	✓	For each variable of interest, give sources of data and measurement methods. Mention if secondary sources such as clinic records were consulted.
	✓	✓	✓	a) How was age ascertained?
	✓	✓	✓	b) How were deaths ascertained?
				How were causes of death ascertained?
	✓	✗	✓	c) How were height (length), weight and oedema measured?
	✗	✓	✓	d) Reference the formulae and indicators used for nutritional prevalence, CMR and U5MR
	✗	✓	✓	e) How was vaccination status determined (card, history, scar?)
Authorization and Ethical Considerations	✓	✓	✓	Was authorization for this survey obtained?
	✗	✗	✗	State whether ethical approval approval was obtained
	✗	✗	✗	Describe the informed consent procedure
Bias	✗	✗	✗	Describe any efforts to address potential sources of bias
Study size	✓	✓	✓	State how the sample size was determined and provide all assumptions. including but not limited to:
	✗	✓	✓	a) What design effect was assumed (cluster survey)?
				b) What CMR (and U5MR) was assumed?
	✗	✓	✓	c) What prevalence of GAM/SAM was assumed?
	✗	✓	✓	d) What degree of precision is desired?
Survey Design	✓	✓	✓	Describe survey sampling design
	✓	✓	✓	a) Describe household selection procedures
	✗	✗	✗	b) Describe procedures to revisit absent households
Survey Teams	✓	✓	✓	Describe training procedures
	✓	✓	✓	State number of surveyors and their degree of professional training
	✗	✓	✓	State how the survey was piloted
Data Accuracy	✗	✓	✓	Describe strategies to ensure data accuracy (e.g., double entry)
Statistical methods	✗	✓	✓	a) Describe all statistical methods
	✗	✗	✗	b) Explain how missing data were addressed
	✗	✓	✓	d) Provide software used for statistical analyses

**Table 4:** Critical review criteria (results and interpretation) and results of three reviewed surveys

	**Survey** **(Ref)**			
	**1** **(14)**	**2** **(15)**	**3** **(16)**	
				**Criteria**

**Results**				

Participants	✓	✓	✓	a) Report number of individuals surveyed
	✓	✓	✓	b) Report non-participation (refusals)
	✓	✓	✓	c) Report number of households surveyed
	✓	✓	✓	d) Give characteristics of survey participants (e.g. demographic, clinical, social)
	✗	✗	✓	e) Indicate number of participants with missing data for each variable of interest
Main Results	✓	✓	✓	Summarize key results with reference to survey objectives
	✓	✓	✓	a) Provide estimates and their precision (eg, 95% confidence interval with design effect if cluster based sampling).
	✗	✗	✗	b) Report causes of death
	✗	✗	✗	c) Report absolute numbers of deaths
	✗	✗	✗	d) Report absolute numbers of other variables of interest
Limitations	✗	✗	✗	Discuss limitations of the survey, taking into account sources of potential bias or imprecision. Discuss both direction and magnitude of any potential bias

**Interpretation**				
Interpretation	✓	✓	✓	Give an overall interpretation of results considering objectives, limitations, results from similar surveys, and other sources of information
Generalisability	✗	✗	✗	Discuss the generalisability (external validity) of the study results
Funding	✓	✓	✓	State the funding source for the survey
Conflict of interest statement	✗	✗	✗	Provide statement concerning conflict of interests and if none, state this.
Follow-up	✗	✗	✓	State to whom these results will be provided

**Recommendations**	✓	✓	✓	Provide recommendations on a course(s) of action based on interpretation of findings

Nonetheless, adherence to reporting standards by itself does not guarantee that useful information will be presented. The three surveys made similar conclusions but commonly failed to provide further qualification of the findings. For example, measles vaccination coverage was uniformly low, in all three surveys - one survey documented that 3.3% of children were vaccinated with card confirmation, with 89% vaccinated according to parental reporting. That study concluded that coverage was sufficient, but neglected to discuss the limitations and assumptions concerning this estimate. Another recommended that the 'health status of the population be improved'; a non-specific recommendation inadequate to guide decision-making. Broader considerations of context were also lacking in the interpretation of findings. For example, nutritional assessments conducted at two different times of the seasonal cycle may have the same result, but have markedly different operational implications. None of the three nutritional assessments we reviewed considered the local seasonality of nutritional status. Appropriate timing of surveys may therefore be another important factor in guiding a meaningful intervention. For one of the surveys, NGO staff were evacuated immediately after the survey as security deteriorated (personal communication with agency). Consequently, the survey results are of limited value. While such events are not always predictable, local circumstances must be considered when planning the allocation of limited resources.

Since field surveys are usually conducted in settings where routine health information systems are absent (such as reporting of births and deaths, communicable and non-communicable surveillance systems), they remain a frequently used and valuable tool for informing interventions. To maximise finite resources and appropriately address health problems during humanitarian crises, it is necessary that surveys using currently accepted methods are well implemented. Further, organisations need to cooperate in developing novel tools suitable for the changing nature of humanitarian crises - for example, there has been a shift towards displaced populations being accommodated by existing host communities and in informal settlements in urban settings rather than in large refugee camps, yet survey methods for mortality and nutritional assessments have barely evolved. Indeed, there may be instances when establishing prospective surveillance systems, however rudimentary, are preferable to the tradition of periodic surveys, such as when organizations are present in an area for an extended period. For all of us involved in humanitarian crises, there is a clear need to reflect on the role and conduct of field surveys and to look beyond the standard methods for measuring mortality and malnutrition.

## Competing interests

All authors, except PS and BC, are employed by organizations which conducted the surveys reviewed in this manuscript.

## Authors' contributions

RFG, FJL, HP, and EG had full access to all the data in the study and take responsibility for the integrity of the data and the accuracy of the data analysis. All authors participated in the conception and design of the study; analysis and interpretation of data; drafting the paper and revising it critically for substantial intellectual content. All authors read and approved the final manuscript.

The Health Nutrition Tracking Service (HNTS) funded this research.
